# Determinants of balance impairment in individuals with Chronic Obstructive Pulmonary Disease: A secondary analysis of a randomized controlled trial

**DOI:** 10.1016/j.rmed.2025.108537

**Published:** 2025-12

**Authors:** Hassan Alrabbaie, Marla Beauchamp, Cindy Ellerton, Roger Goldstein, Annemarie L. Lee, Jennifer A. Alison, Gail Dechman, Kimberley J. Haines, Samantha L. Harrison, Anne E. Holland, Alda Marques, Lissa Spencer, Michael K. Stickland, Elizabeth H. Skinner, Dina Brooks

**Affiliations:** a School of Rehabilitation Sciences, Faculty of Health Science, McMaster University, Canada; b Department of Rehabilitation Sciences, Faculty of Applied Medical Sciences, Jordan University of Science and Technology, Irbid, Jordan; c Department of Respiratory Medicine, West Park Healthcare Centre, Toronto, Canada; d Department of Physical Therapy, Faculty of Medicine, University of Toronto, Toronto, ON, Canada; e Rehabilitation Sciences Institute, School of Graduate Studies, University of Toronto, Toronto, ON, Canada; f Institute for Breathing and Sleep, Melbourne, VIC, Australia; g Department of Physiotherapy, School of Primary and Allied Health Care, Monash University, Melbourne, VIC, Australia; h School of Health Sciences, Faculty of Medicine and Health, University of Sydney, Sydney, NSW, Australia; i Allied Health, Sydney Local Health District, Sydney, Australia; j School of Physiotherapy, Faculty of Health, Dalhousie University, Halifax, NS, Canada; k Physiotherapy Department, Western Health, Department of Critical Care, The University of Melbourne, Melbourne, VIC, Australia; l School of Health and Life Sciences, Teesside University, Middlesbrough, UK; m Department of Physiotherapy, Alfred Health, Melbourne, VIC, Australia; n Respiratory Research, Monash University, Melbourne, VIC, Australia; o Lab3R-Respiratory Research and Rehabilitation Laboratory, School of Health Sciences (ESSUA), Institute of Biomedicine (iBiMED), University of Aveiro, Aveiro, Portugal; p Department of Physiotherapy, Royal Prince Alfred Hospital, Camperdown, NSW, Australia; q Division of Pulmonary Medicine, Faculty of Medicine and Dentistry, University of Alberta, Edmonton, AB, Canada; r G.F. MacDonald Centre for Lung Health, Covenant Health, Edmonton, AB, Canada

**Keywords:** COPD, Balance, Falls, Dyspnea, Functional performance, Exercise capacity

## Abstract

**Background:**

Balance impairment is common in individuals with Chronic Obstructive Pulmonary Disease (COPD), increasing fall risk and reducing functional independence. This study investigated functional, clinical, and demographic determinants of balance performance in individuals with COPD.

**Methods:**

This secondary analysis included participants from a randomized controlled trial involving pulmonary rehabilitation centers. Balance was evaluated using the Berg Balance Scale (BBS) and Balance Evaluation Systems Test (BESTest). Functional assessments comprised the 30-Second Chair Stand Test (30s CST) and Six-Minute Walk Test (6-MWT). Clinical variables included lung function, dyspnea, comorbidities, medications, fall history, supplemental oxygen, and gait aid use. Multiple regression analyses were conducted to evaluate associations between balance, functional, clinical, and demographic factors.

**Results:**

Of the 244 participants (mean age: 71 ± 9 years; 57 % male), 87 % reported balance deficits and 50 % had at least one fall in the past year. Functional capacity was strongly correlated with balance scores (r > 0.5, p < 0.001). Clinical factors, including dyspnea, comorbidities, gait aid, and supplemental oxygen use, were also significant. The full regression model explained 58 % of the variance in BESTest scores and 51 % in BBS scores. Each additional 30s CST repetition predicted a 1.22-point increase in BESTest scores and a 0.59-point increase in BBS scores. Less severe dyspnea was associated with higher scores on both balance measures.

**Conclusion:**

Balance performance in individuals with COPD is influenced by both clinical and functional parameters. Identification of these factors supports the development of targeted interventions to address balance impairment and improve patient outcomes in this population.

## Highlights

• Lower limb muscle strength and exercise tolerance independently predict balance in COPD.
• Dyspnea severity is significantly associated with impaired balance performance.
• Use of supplemental oxygen and gait aids reflects a higher risk of balance deficits and falls.
• Pulmonary function (FEV_1_) is not an independent predictor of balance impairment.

## 1 Introduction

Chronic Obstructive Pulmonary Disease (COPD) is a progressive and complex respiratory condition that significantly affects individuals’ quality of life and imposes a substantial burden on healthcare systems [1]. While the hallmark features of COPD are respiratory symptoms, the disease is also associated with a broad spectrum of systemic and non-respiratory complications, including pain, depression, anxiety, sleep disturbances, and cognitive decline [2,3]. These comorbidities collectively contribute to reduced functional capacity, restricted daily activities, and exacerbate the overall disease burden [4].

Among the non-respiratory complications of COPD, balance impairment has emerged as a critical concern [5,6]. Balance impairment is linked to limited mobility and independence, adversely affecting health-related quality of life [[5], [6], [7], [8], [9]]. Pichon et al. [10] reported that the symptoms subscore of the St. George's Respiratory Questionnaire was strongly associated with balance deficits in COPD, emphasizing the contribution of respiratory symptoms, such as dyspnea, to impaired balance and their broader impact on daily function and well-being. Importantly, impaired balance is associated with an increased risk of falls, which can lead to injuries, hospitalization, functional decline, and greater healthcare utilization [3,[11], [12], [13], [14]]. Compared to age-matched healthy controls, individuals with COPD exhibit poorer balance and have a 55 % higher likelihood of falling [[15], [16], [17]].

Although prior studies have investigated the determinants of balance impairment in individuals with COPD [[18], [19], [20], [21], [22], [23], [24], [25]], to date, few studies have comprehensively evaluated both pulmonary and extrapulmonary contributors using validated and multidimensional assessment tools [5,7]. Furthermore, small sample sizes or single-site designs limit most existing studies, and the role of potentially modifiable determinants, such as lower limb muscle strength, exercise tolerance, and dyspnea, remains insufficiently clarified [5].

Therefore, the present study aimed to investigate the functional, clinical, and demographic factors that contribute to balance performance in individuals with COPD. Specifically, our objectives were:

1 To describe the association between functional capacity, specifically lower body strength, exercise tolerance, and balance performance in individuals with COPD.
2 To assess the association between clinical indicators, including lung function, dyspnea, comorbidities, supplemental oxygen use, gait aid use, medication use, and history of falls, with balance performance.
3 To evaluate the association between demographic and anthropometric factors (age, sex, smoking history, and body mass index (BMI)) and balance performance in individuals with COPD.

We hypothesized that functional, clinical, and demographic factors would all be significantly associated with balance impairments in individuals with COPD.

## 2 Methods

### 2.1 Study design

This was a secondary analysis of baseline data collected as part of an international multicenter randomized controlled trial (RCT) of pulmonary rehabilitation (PR) with balance training for fall reduction in COPD [26]. The primary RCT evaluated the impact of adding balance training to standard PR on fall outcomes among individuals with COPD who were at high risk of falling [26].

The trial was conducted between 2017 and 2021 across nine outpatient PR centers in Canada, Australia, Portugal, and the United Kingdom. The full study protocol and results were published [26], and the trial was prospectively registered at ClinicalTrials.gov https://clinicaltrials.gov/study/NCT02995681 [27]. The RCT demonstrated that while balance training improved certain balance measures post-intervention, it did not reduce the incidence of falls over a 12-month follow-up [26]. Ethical approval for the RCT was granted by all participating institutional research ethics boards (**se**e Supplementary Table 1), and written informed consent was obtained from all participants before data collection.

### 2.2 Participants

A total of 244 participants with COPD who completed the baseline assessment in the RCT were included in this analysis. Inclusion criteria were: (1) diagnosis of COPD according to the Global Initiative for Chronic Lung Disease COPD (GOLD) criteria, defined as post-bronchodilator forced expiratory volume in 1 s (FEV_1_)/forced vital capacity (FVC) ratio <0.70 [28]; (2) ability to provide informed consent; (3) completion of baseline balance assessment, including the Berg Balance Scale (BBS) [29] and the Balance Evaluation Systems Test (BESTest) [30]; and (4) high fall risk, defined as self-reported balance deficits, a fall history within the last two years, or a recent near fall.

Individuals were excluded if they met any of the following criteria: (1) inability to communicate due to language barrier; (2) hearing or cognitive impairment (e.g., dementia or neurological condition); or (3) neurological or musculoskeletal condition that severely limited mobility and postural control (e.g., cerebrovascular accident, Parkinson's diseases, or lower limb amputee).

Participants enrolled in the RCT were assessed at baseline (enrolment in PR and the study), upon completion of PR, and at the end of the 12-month follow-up period. Demographic and clinical characteristics, including age, sex, BMI, smoking history, history of falls in the previous one and two years, presence of comorbidities, polypharmacy, supplemental oxygen use, gait aid use, dyspnea scores, and measures of physical function and balance were collected at baseline. Dyspnea severity was measured using the Baseline Dyspnea Index (BDI), with higher scores indicating less dyspnea. Comorbidities were self-reported physician-diagnosed conditions, documented as both a list and count. Medication use was recorded by the number of prescribed drugs. Gait aid use was categorized as none or rollator use.

### 2.3 Outcome measures

Clinical outcome measures were collected at baseline to assess participants’ functional status and clinical characteristics. These are summarized in Table 1 and include validated tools that assess balance, functional capacity, respiratory function, and dyspnea.

**Table 1:** Baseline outcome measures and their constructs.

Construct	Measure	Measure description
**Balance**	**BBS**	Includes 14 items that evaluate specific functional tasks, such as reaching, single-leg standing, turning around, and transfers. Each item is scored from 0 to 4, with a maximum score of 56. Higher scores indicate better balance performance [29]. A score of 46 or lower has been found useful for identifying individuals at risk of falls. [31]. The BBS has demonstrated good reliability (ICC = 0.94) and moderate to strong validity (r = 0.53–0.75) in evaluating balance control in older adults and the COPD clinical population [32]. MCID for the BBS is a change of 5–7 points [33], while the MDC has been reported as 3.5 to 5.9 points [32,34].
	**BESTest**	Includes 36 items that evaluate six subsystems of balance control: biomechanical, stability limits/verticality, anticipatory postural adjustments, automatic postural adjustments, sensory orientation, and dynamic balance during gait. Each item is scored from 0 to 3 (worst to best performance). Total scores range from 0 % to 100 %, and a change of 13–17 points on the BESTest is considered clinically significant in patients with COPD [30]. The MCD has been established at 6.3 points [32]. A cutoff score of 67 %, equivalent to 82 points, has been identified as optimal for identifying individuals at risk of falls. [35]. The BESTest is a valid (r = 0.64) and reliable (ICC = 0.85) measure of balance control [32].
**Lower body strength**	**30s CST**	Measures the total number of complete stands performed within 30 s, starting from a seated position and returning to it between each repetition. Higher scores indicate greater lower extremity strength [36].
**Exercise tolerance**	**6-MWT**	Measures the total distance walked (meters) in 6 min [37]. A greater distance walked reflects a greater exercise capacity [38]. The 6-MWT demonstrated excellent intra- and inter-rater reliability in patients with COPD [39].
**Lung function**	**Spirometry**	Gold standard for classification of disease severity based on FEV_1_ [28].
**Dyspnea**	**BDI**	Includes a three-part measurement of the experience of dyspnea during daily activities [40]. It evaluates functional impairment, task magnitude, and effort magnitude. Each category is scored from 0 to 4 (severe to no impairment), with the total score ranging from 0 to 12, where a score of 12 corresponds to no dyspnea [41]. A 1-unit change in the BDI is generally considered the MCID in COPD [40].

Abbreviations: BDI: baseline dyspnea index; BBS: Berg Balance Scale; BESTest: Balance Evaluation Systems Test; FEV_1_: forced expiratory volume in 1 s; MCID: minimal clinically important change; MDC: minimal detectable change; 6-MWT: Six-Minute Walk Test; 30s CST: 30-s repeated chair stand test.

A detailed overview of the BBS and BESTest is provided in Supplementary Table 3.

## 3 Data analysis

All statistical analyses were performed using IBM SPSS Statistics version 27 (IBM Corp., Armonk, New York) [42]. Descriptive statistics were computed for all study variables. Continuous variables (e.g., age, FEV_1_, 6-MWT, 30s CST, BDI total score, BBS total score, BESTest total score) were summarized as mean and standard deviation (mean ± SD). Categorical variables (e.g., sex, oxygen use, gait aid use, and fall history) were presented as frequencies and percentages.

The normality of continuous variables was assessed using the Shapiro-Wilk test and confirmed by visual inspection of histograms. Pearson correlation coefficients were used to examine the bivariate association between balance scores (BBS and BESTest) and potential predictor variables. Correlation strengths were interpreted as follows: 0.1 ≤ r < 0.3 indicates a weak association; 0.3 ≤ r < 0.5 represents a moderate association; and r ≥ 0.5 signifies a strong association [43].

Multiple linear regression analyses examined the association between independent variables and balance performance. Predictor selection was informed by prior literature and clinical relevance. Separate models were constructed for: (1) functional factors (30s CST and 6-MWT); (2) clinical factors (including FEV_1_, dyspnea (BDI score), number of comorbidities, oxygen use, gait aid use, medication count, and fall history); (3) demographic and anthropometric factors (age, sex, BMI, and smoking history). All variables within each domain were entered simultaneously within respective models. A comprehensive model including all predictors from three domains was also tested to evaluate their combined association with balance outcomes. Results were reported as unstandardized regression coefficients (B), standard errors (SE), standardized coefficients (β), and p-values. Model fit was described with R^2^, adjusted R^2^, and F-statistics. Statistical significance was set at p < .05. Multicollinearity was monitored using variance inflation factors (VIF), with VIF values below 2 considered acceptable, indicating no significant multicollinearity.

To enhance the robustness and reliability of our regression estimates, stratified bootstrap resampling with 1000 iterations was performed, incorporating oxygen use and gait aid use as stratification variables to preserve their respective subgroup proportion.

## 4 Results

### 4.1 Demographic, clinical, and functional characteristics

A total of 244 participants with COPD were included in the analysis, with 57 % identifying as male. The mean age was 71 ± 9 years, and the mean BMI was 27.5 ± 6.5 kg/m^2^. Participants reported a mean smoking history of 45.5 ± 37.3 pack-years. Supplemental oxygen use was reported by 16 % of the participants, and 13 % used a gait aid for mobility. A total of 87 % reported balance deficits, 50 % reported at least one fall in the past year, and 60 % had fallen within the past two years.

The mean FEV_1_ was 1.5 ± 0.7 L, corresponding to 50 ± 19 % of predicted values, consistent with moderate to severe airflow limitation. Mean balance assessment scores were 47.9 ± 8.3 on the BBS and 75.8 ± 18.9 on the BESTest. Functional performance was reflected by a mean of 9.4 ± 5.1 repetitions on 30s CST and 309 ± 153 m on the 6-MWT (see Table 2).

**Table 2:** Clinical and functional characteristics (N = 244).

Clinical Characteristics	n (%)	mean (SD)
**Age (years)**		71 (9)
**Sex**		
Male	138 (57)	
**BMI (kg/m^2^)**		27.5 (6.5)
**Smoking history (pack years)**		45.5 (37.3)
**Use of Supplementary Oxygen**	39 (16)	
**Use of Gait Aid**	32 (13)	
**Self-report balance deficits**	212 (87)	
**Fall history last year**	121 (50)	
**Fall history last two years**	147 (60)	
**Total comorbidities**		5.4 (2.7)
**Total medications**		6.9 (3.3)
**Pulmonary Function**		
FEV_1_ (L)		1.5 (0.7)
FEV_1_ Predicted (%)		50.1 (19.4)
FVC (L)		2.5 (0.9)
FVC Predicted (%)		73.8 (21.8)
FEV_1_/FVC		0.6 (0.5)
**Balance Measures**		
BESTest		75.8 (18.9)
BBS		47.9 (8.3)
**Functional Measures**		
BDI		6.1 (2.7)
30s CST		9.4 (5.1)
6MWT (meters)		308.6 (153.1)

Abbreviations: N: sample size; SD: standard deviation; BMI: body mass index; COPD: chronic obstructive pulmonary disease; BDI: baseline dyspnea index; FEV_1_: forced expiratory volume in 1 s; FVC: forced vital capacity; BESTest: Balance Evaluation Systems Test; BBS: Berg Balance Scale; 30s CST: 30-s Repeated Chair Stand Test; 6-MWT: Six-Minute Walk Test.

### 4.2 Associations with balance performance

Better functional performance was associated with higher balance scores on both the BBS and the BESTest. Greater lower limb strength, as measured by the 30s CST, was strongly positively associated with higher balance scores (BBS: *r* = 0.578, *p* < .001; BESTest: *r* = 0.596, *p* < .001). Similarly, longer walking distance (6-MWT), indicating better exercise tolerance, was significantly associated with better balance performance (BBS: *r* = 0.519, *p* < .001; BESTest: *r* = 0.591, *p* < .001).

Among clinical factors, less dyspnea (higher BDI scores) was moderately associated with better balance (BBS: *r* = 0.444, *p* < .001; BESTest: *r* = 0.504, *p* < .001). A greater number of comorbidities was associated with lower balance scores (BBS: *r* = - 0.213, *p* < .001; BESTest: *r* = - 0.296, *p* < .001). Use of a gait aid showed a moderate negative association with balance, with poorer scores among users (BBS: *r* = - 0.293, *p* < .001; BESTest: *r* = - 0.321, *p* < .001). A history of falls in the previous year was also associated with lower balance performance (BBS: r = - 0.174, *p* < .001; BESTest: *r* = - 0.230, *p* < .001). In contrast, FEV_1_ was not significantly associated with balance outcomes.

For demographic and anthropometric factors, older age demonstrated a significant negative association with balance (BBS: *r* = - 0.264; BESTest: *r* = - 0.318; both p < .001). Female sex had a weak negative association (BBS: *r* = - 0.127; BESTest: *r* = - 0.192). No significant associations were observed for BMI or smoking history. Full correlation results are presented in Table 3.

**Table 3:** Correlation summary of factors associated with balance impairment.

Factors	BBS scores (*r*)	BESTest scores *(r)*
30s CST	.578**	.596**
6-MWT (meters)	.519**	.591**
FEV_1_	.027	.088
Dyspnea (BDI)	.444**	.504**
Total comorbidities	−.213**	−.296**
Total medications	−.116	−.222**
Oxygen use	.041	−.034
Gait aid use	−.293**	−.321**
Fall history last year	−.174**	−.230**
Age	−.264**	−.318**
Sex	−.127*	−.192**
BMI	−.059	−.046
Smoking history	.001	−.026

N = 244. BBS: Berg Balance Scale, BESTest: Balance Evaluation Systems Test, FEV_1_: forced expiratory volume in 1 s, BDI: Baseline Dyspnea Index, BMI: body mass index, 30s CST: 30-s Repeated Chair Stand Test, 6-MWT: Six-Minute Walk Test.

**p < .001, *P < .05 (two-tailed).

### 4.3 Predictors of balance performance

#### 4.3.1 Functional model

The functional regression model significantly predicted balance performance, explaining 41 % of the variance in BBS [F (2, 241) = 84.9, R^2^ = 0.41, *p* < .001] and 48 % in BESTest [F (2, 241) = 111.6, R^2^ = 0.48, *p* < .001] (Table 4). Each additional repetition in the 30s CST was associated with a 0.69-point increase in BBS [B (SE) = 0.69 (0.09), *p* < .001] and a 1.52-point rise in BESTest total scores [B (SE) = 1.52 (0.2), *p* < .001]. Similarly, greater distance walked on the 6-MWT predicted higher balance scores, with each additional meter associated with a 0.02-point increase in BBS scores [B (SE) = 0.02 (0.003), *p* < .001] and a 0.05-point rise in BESTest total scores [B (SE) = 0.05 (0.01), *p* < .001].

**Table 4:** Summary of regression models for balance measures.

Models	BBS					BESTest			
	**Variable**	**B**	**SE**	**β**	***p***	**B**	**SE**	**β**	***p***
**Functional impairment**	30s CST	.69	.09	.43	<.001**	1.52	.20	.41	<.001**
	6-MWT (meters)	.02	.003	.32	<.001**	.05	.01	.40	<.001**
	R^2^ = .41 Adj R^2^ = .41 Overall *F (2,241) =84.9* *P*-value <.001					R^2^ = .48 Adj R^2^ = .48 Overall *F (2,241) =111.6* *P*-value <.001			
**Clinical factors**	FEV_1_	−.06	.17	−.018	.74	.22	.36	.03	.55
	Dyspnea (BDI)	1.29	.17	.42	<.001**	3.12	.37	.45	<.001**
	Total comorbidities	−.53	.18	−.17	.004*	−1.31	.39	−.19	<.001**
	Total medications	.13	.15	.05	.41	−.16	.33	−.03	.62
	Oxygen use	3.49	1.27	.16	.006*	5.37	2.74	.10	.052
	Gait aid use	−5.44	1.38	−.22	<.001**	−12.32	2.96	−.22	<.001**
	Fall history last year	−1.58	.92	−.09	.09	−5.09	1.98	−.13	.01*
	R^2^ = .31 Adj R^2^ = .27 Overall *F (7.236) = 14.9* *P*-value <.001					R^2^ = .38 Adj R^2^ = .37 Overall *F (7,236) =20.1* *P*-value <.001			
**Demographic and anthropometric factors**	Age	−.25	.06	−.28	<.001**	−.68	.12	−.33	<.001**
	Sex	−2.35	1.05	−.14	.03*	−8.19	2.32	−.21	<.001**
	BMI	−.08	.08	−.06	.34	−.12	.18	−.04	.49
	Smoking history	−.002	.01	−.01	.87	−.01	.03	−.02	.77
	R^2^ = .10 Adj R^2^ = .08 Overall *F (4,239) = 6.21* *P*-value <.001					R^2^ = .15 Adj R^2^ = .14 Overall F (4,239) *= 10.47* *P*-value <.001			
**Full model**	30s CST	.59	0.09	.37	<.001**	1.22	.19	.33	<.001**
	6-MWT (meters)	.02	.003	.29	<.001**	.04	.01	.28	<.001**
	FEV_1_	−.24	.15	−.08	.10	−.25	.31	−04	.42
	Dyspnea (BDI)	.58	.17	.19	.001*	1.55	.36	.22	<.001**
	Total comorbidities	−.09	.17	−.03	.58	−.28	.35	−.04	.43
	Total medications	.15	.13	.06	.25	−.13	.28	−.02	.65
	Oxygen use	3.38	1.1	.15	.002*	5.52	2.33	.11	.02*
	Gait aid use	−4.25	1.21	−.17	.001*	−9.04	2.56	−.16	.001*
	Fall history last year	−.87	.80	−.05	.28	−3.17	1.71	−.08	.07
	Age	−.01	.05	−.02	.77	−.14	.10	−.07	.18
	Sex	.87	.85	.05	.31	−.30	1.81	−.01	.87
	BMI	−.04	.06	−.03	.51	−.05	.13	−.02	.73
	Smoking history	.02	.01	.09	.43	.04	.02	.08	.09
	R^2^ = .51 Adj R^2^ = .48 Overall *F (13,230) = 18.39* *P*-value <.001					R^2^ = .58 Adj R^2^ = .55 Overall *F (13,230) =24.05* *P*-value <.001			

*P < 0.05, **P < 0.01 (N = 244); *B:* unstandardized coefficient, *SE*: standard error, *β*: standardized coefficient, BBS: Berg Balance Scale, BESTest: Balance Evaluation Systems Test, FEV_1_: forced expiratory volume in 1 s, BDI: Baseline Dyspnea Index, BMI: body mass index, 30s CST: 30-s Repeated Chair Stand Test, 6-MWT: Six-Minute Walk Test.

#### 4.3.2 Clinical model

In the clinical factors model, dyspnea, comorbidity count, and gait aid use emerged as significant contributors to balance performance. The model explained 31 % of the variance in BBS scores [F (7,236) = 14.9, R2 = 0.31, *p* < .001] and 38 % of the variance in the BESTest total scores [F (7,236) = 20.1, R2 = 0.38, *p* < .001]. A higher BDI score, which indicates less severe dyspnea, predicted better balance, with a 1.29-point increase in BBS [B (SE) = 1.29 (0.17), *p* < .001] and a 3.12-point increase in BESTest total scores [B (SE) = 3.12 (0.37), *p* < .001] per point.

Conversely, an additional comorbidity was associated with a reduction in BBS [B (SE) = - 0.53 (0.18), *p* = .004] and BESTest total scores [B (SE) = - 1.31 (0.39), *P* = .001]. Use of a gait aid was linked to lower BBS [B (SE) = - 5.44 (1.38), *p* < .001] and BESTest total scores [B (SE) = −12.32 (2.96), *p* < .001]. Supplemental oxygen use predicted a 3.49-point increase in BBS [B (SE) = 3.54 (1.27), *p* < .001]; however, its effect on BESTest total scores was not significant [B (SE) = 5.37 (2.74), *p* = .052].

History of a fall in the previous year significantly predicted lower BESTest total scores [B (SE) = - 5.09 (1.98), *p* = .01] but not BBS scores [B (SE) = - 1.58 (0.92), *p* = .09]. Neither medication count nor FEV_1_ was a significant predictor of balance performance in either measure (Table 4).

#### 4.3.3 Demographic and anthropometric model

The demographic and anthropometric model, including age, sex, smoking history, and BMI, explained 10 % of variance in the BBS scores [F (4,239) = 6.21, R^*2*^ = 0.10, *p* < .001] and 15 % in BESTest total scores [F (4,239) = 10.47, R^*2*^ 0.15, *p* < .001]. Older age was associated with poorer balance performance, with each additional year of age associated with a 0.25-point decrease in BBS [B (SE) = - 0.25 (0.06), *p* < .001] and a 0.68-point decrease in BESTest total scores [B (SE) = - 0.68 (0.12), *p* < .001].

Female participants had lower balance scores compared to males, with a 2.35-point reduction in the BBS scores [B (SE) = −2.35 (1.05), p = .03] and an 8.19-point reduction in BESTest total scores [B (SE) = −8.19 (2.32), p < .001]. Smoking history and BMI were not significant predictors of balance performance in either measure (*p* > .05) (Table 4).

#### 4.3.4 Full model

In the comprehensive model including all predictors, 51 % of the variance in BBS scores [F *(*13, 230) = 18.39, R^2^ = 0.51, *p* < .001] and 58 % in BESTest scores [F (13, 230) = 24.05, R^2^ = 0.58, *p < .*001] was explained. Independent predictors of balance performance across both outcomes included higher 30s CST and 6-MWT scores, higher BDI scores, oxygen use, and gait aid use. Age, sex, total comorbidities, and history of falls were not retained in the final model. (Table 4). Stratified bootstrap resampling (1000 iterations) confirmed the stability of these associations with confidence intervals consistent with the conventional estimates (Supplementary Table 2).

## 5 Discussion

This study provides a comprehensive assessment of functional, clinical, and demographic factors associated with balance performance among individuals with COPD. By systematically comparing models focused on these factors, we identified exercise tolerance, lower limb strength, dyspnea burden, comorbidity count, supplemental oxygen use, use of a gait aid, fall history, age, and sex as factors significantly associated with balance impairment in domain-specific analyses. However, in the multivariable model adjusting for all domains, only functional performance (6-MWT, 30s CST), dyspnea severity (BDI score), supplemental oxygen use, and use of a gait aid were independently associated with balance impairment. Notably, comorbidity burden, fall history, age, and sex were not retained in the final model, suggesting their influence is mediated through functional and clinical variables. These findings highlight that balance impairment in individuals with COPD is influenced predominantly by potentially modifiable functional factors, rather than by demographic characteristics or markers of disease severity.

Our findings highlight the central role of functional performance, particularly exercise tolerance and lower limb muscle strength, in balance impairments among individuals with COPD [18,20,44]. While prior studies have suggested this link, our analysis demonstrates that these functional factors remain independent contributors even after adjustment for clinical and demographic variables. Consistent with prior studies, poorer performance on the 6-MWT and reduced lower limb strength were associated with lower balance performance [5,18,20,44]. Loughran et al. [5] in a meta-analysis, reported that balance deficits in COPD are largely driven by muscle weakness, physical inactivity, and limited exercise capacity. COPD-related alterations in both respiratory and peripheral muscle function contribute to fatigue and functional decline, increasing long-term morbidity and mortality [45,46]. The 6-MWT and the 30s CST, reflecting lower limb function and endurance, are valuable tools for identifying individuals at risk of balance impairment and subsequent adverse mobility outcomes [47,48]. These findings emphasize that lower limb muscle weakness is not only linked to reduced mobility but also to increased fall risk, as muscle impairment directly impacts postural control and stability [5,7,49]. Therefore, balance assessment in individuals with COPD should routinely incorporate functional performance metrics, such as 6-MWT and standardized strength tests [50], to guide personalized interventions aimed at improving both balance and broader physical function in this population.

Airflow obstruction, as measured by FEV_1,_ was not significantly associated with balance performance, even in models restricted to clinical predictors. This finding aligns with previous studies, which also failed to identify a significant relationship between FEV_1_ and balance [18,25,31,51]. The association between FEV_1_ and balance is frequently attributed to the systemic effects of COPD, including muscle weakness, deconditioning, and hypoxemia, all of which are themselves influenced by disease severity [52,53]. Consistent with the current results, other studies have demonstrated that FEV_1_ is a weaker correlate, serving more as an indirect marker rather than as a direct predictor [18,25,31,51]. In individuals with moderate to severe COPD, extrapulmonary manifestations such as muscle dysfunction and dyspnea become the dominant determinants of balance [18,20,31]. This complexity likely reflects the multifactorial interplay where FEV_1_ functions as an indirect marker of disease burden, while functional tests more accurately capture the direct impact on mobility and postural stability [51].

Our cohort, with a mean FEV_1_ of 50.1 % predicted, falls within the moderate to severe classification of COPD [54]. Within this range, extrapulmonary features, such as muscle dysfunction and dyspnea, become more dominant determinants of balance than FEV_1_ itself [18,20,31,51]. Furthermore, systemic inflammation and hypoxia, which may manifest independently of FEV_1_ values, can compromise central nervous system function, disrupting motor control and sensory integration essential for balance [25,53,55,56]. Thus, while FEV_1_ reflects the degree of airway obstruction, it does not fully capture these broader systemic effects on balance. For example, de Castro et al. [57] identified an association between total lung capacity and static balance, pointing to the role of hyperinflation and altered thoracic mechanics in postural stability. In contrast, Ucgun et al. [18] suggested that better walking distance and lower dyspnea may have indicated a lower prevalence of hyperinflation in their cohort. The absence of FEV_1_ association in our sample may be explained by the relatively greater impact of extrapulmonary factors, particularly muscle strength and endurance, on balance among individuals with COPD [5].

Less dyspnea, indicated by higher BDI scores, was independently associated with better balance. This aligns with prior evidence showing that greater dyspnea is associated with poorer balance, even after adjusting for muscle strength and physical performance [58]. Dyspnea also contributes to reduced physical activity, which accelerates muscle deconditioning and impairs overall function [59]. These findings reinforce dyspnea's dual role as both a direct contributor to balance impairment and a marker of broader physical decline.

An unexpected finding was the positive association between supplemental oxygen use and balance performance. This contrasts with previous studies reporting that oxygen use is associated with poorer balance and functional outcomes in COPD [31,55,60]. For instance, Butcher et al. [60] and Park et al. [55] observed worse performance among oxygen users, attributing this to greater disease severity. Conversely, Oliveira et al. [61] found that oxygen use was independently related to lower fall incidence over 12 months, possibly due to self-imposed restriction of physical activity, which reduces exposure to hazards. While supplemental oxygen can enhance certain functional outcomes, such as exercise endurance [62], its use generally signals more advanced disease and is not typically associated with improving balance [20,60]. This paradox is critical as underlying hypoxemia may persist despite oxygen therapy, continuing to impair postural control [25,31]. Inadequate oxygenation directly affects central nervous system function, resulting in reduced coordination, delayed responses, and impaired sensory processing, all of which are essential for maintaining balance [25,55,56].

Building on these physiological insights, our findings suggest that the need for supplemental oxygen is more accurately interpreted as a marker of disease severity than a factor directly enhancing balance. Their oxygen dependency may reflect broader systemic dysfunction, warranting individualized assessment and interventions. This underscores the need for comprehensive multi-dimensional evaluations that consider clinical and behavioral contributors to balance. Future research should explore whether oxygen use has an independent effect on balance beyond its role as a severity marker. Investigating characteristics such as the degree of hypoxemia, duration of oxygen use, and adherence to therapy will help clarify these relationships. Such details are vital for identifying potential confounding factors or unique physiological profiles that may explain conflicting findings and inform precision-based interventions.

Our study also demonstrated a significant negative association between gait aid use and balance performance. This is consistent with the literature, which suggests that assistive device use reflects pre-existing impairments in balance and mobility rather than being a direct cause of functional limitation [19,63,64]. For example, Nguyen et al. [63] found that individuals using gait aids consistently performed worse across several balance tests, including the Timed Up and Go (TUG), TUG Dual-Task, Brief BESTest, and Single Leg Stance (SLS). The repeated association between gait aid use and fall risk further supports the interpretation that assistive device use is a proxy for more severe impairment [63]. While some studies suggest that gait aid use may better predict fall risk than balance scores alone, the overarching evidence positions it as a clinical indicator of compromised mobility in individuals with COPD [13,19,64].

Age, sex, comorbidity burden, and history of falls were associated with balance performance in univariate or domain-specific models but lost significance in the full model. This pattern suggests that their influence is mediated through functional and clinical variables, reinforcing the central role of modifiable factors in COPD-related balance impairment. This mirrors previous research [10,16,22,24,44,65]. For instance, Jacome et al. [24], found that although sex, oxygen use, and airflow limitation were associated with balance impairment in univariate analyses, none remained significant in multivariate models. These findings highlight the importance of targeting modifiable physiological impairments, rather than relying solely on demographic or static clinical indicators, in efforts to improve balance and reduce fall risk in individuals with COPD.

### 5.1 Strengths and limitations

This study has several strengths. First, the use of both the BBS and BESTest provided a multidimensional evaluation of balance, capturing diverse aspects of postural control. Second, the inclusion of a broad set of clinical, functional, and demographic variables enabled a comprehensive analysis of factors contributing to balance impairments. Third, the multicenter study including nine PR centers across Canada, Australia, and Europe enhances the sample diversity, improving the external validity and generalizability of our results. Finally, the relatively large and diverse sample size, compared to many earlier studies, improved statistical power and the robustness of findings. The integration of objective performance metrics and patient-reported outcomes strengthens the clinical relevance of the results.

However, limitations must be acknowledged. The cross-sectional design prevents conclusions about causality and does not allow assessment of changes in balance over time. Longitudinal studies are necessary to address this gap. Additionally, the BBS may exhibit a ceiling effect in higher-functioning individuals, potentially underestimating subtle balance deficits. Although we included a wide range of variables, unmeasured factors, such as cognitive status, type of comorbidities, and medication classes, warrant further exploration as they may influence balance and falls, and should be considered in future research.

## 6 Conclusion

The findings of this study have important clinical implications for managing balance impairment in individuals with COPD. Recognizing the multifactorial nature of such impairments, the balance assessment for those referred to PR should include measures of both physical and respiratory function. Identifying modifiable contributors will support more effective, individualized intervention strategies to improve balance and quality of life in this population.

## CRediT authorship contribution statement

**Hassan Alrabbaie:** Writing – review & editing, Writing – original draft, Visualization, Methodology, Formal analysis, Conceptualization. **Marla Beauchamp:** Writing – review & editing, Validation, Funding acquisition. **Cindy Ellerton:** Writing – review & editing, Project administration, Data curation. **Roger Goldstein:** Writing – review & editing, Validation, Funding acquisition. **Annemarie L. Lee:** Writing – review & editing, Investigation, Funding acquisition, Data curation. **Jennifer A. Alison:** Writing – review & editing, Project administration, Investigation, Funding acquisition, Data curation. **Gail Dechman:** Writing – review & editing, Investigation, Funding acquisition, Data curation. **Kimberley J. Haines:** Writing – review & editing, Investigation, Data curation. **Samantha L. Harrison:** Writing – review & editing, Investigation, Funding acquisition, Data curation. **Anne E. Holland:** Writing – review & editing, Investigation, Funding acquisition, Data curation. **Alda Marques:** Writing – review & editing, Investigation, Funding acquisition, Data curation. **Lissa Spencer:** Writing – review & editing, Investigation, Funding acquisition, Data curation. **Michael K. Stickland:** Writing – review & editing, Investigation, Funding acquisition, Data curation. **Elizabeth H. Skinner:** Writing – review & editing, Investigation, Funding acquisition, Data curation. **Dina Brooks:** Validation, Supervision, Funding acquisition.

## Funding

Funding for this study was provided by the Canadian Institutes for Health Research (CIHR). Marla K Beauchamp was supported by an Emerging Research Leader Initiative from the Canadian Respiratory Research Network, an Early Researcher Award from Ministry of Colleges and Universities of Ontario, and a Canada Research Chair in Mobility, Aging, and Chronic Disease. Dina Brooks was supported by a Canada Research Chair in Rehabilitation for Chronic Obstructive Pulmonary Disease (2008–2018) and a National Sanitarium Association Chair in Respiratory Rehabilitation (2019–2024) Samantha L Harrison is funded by the NIHR [Advanced fellow NIHR300856}. Alda Marques’ research is also supported by national funds through FCT - Fundação para a Ciência e Tecnologia, I.P., under UID 4501- Institute of Biomedicina - Aveiro.

## Declaration of competing interest

The authors declare that they have no competing financial interests or personal relationships that could have influenced the work reported in this paper.
