# Supplementary material for: Determinants of balance impairment in individuals with Chronic Obstructive Pulmonary Disease: A secondary analysis of a randomized controlled trial
Source: Respir Med. 2025 Dec;250:108537. doi: 10.1016/j.rmed.2025.108537 (PMC12706577; doi:10.1016/j.rmed.2025.108537)
Supplement: Multimedia component 1 [file mmc1.docx]

**Table 1: Ethical approval**

| **Committee name** | **County** | **Approval number** |
| --- | --- | --- |
| Joint West Park Healthcare Centre/Toronto Central Community Care Access Centre/Toronto Grace Health Centre Research Ethics Board | Canada | 16-018-WP |
| Nova Scotia Health Authority Research Ethics Board | Canada | 1021990 |
| University of Alberta Research Ethics Board | Canada | 1921, Pro00069642 |
| University of British Columbia/Providence Health Care Research Ethics Board | Canada | H16-02876 |
| University of Toronto Research Ethics Board | Canada | 33891 |
| Alfred Health Research Ethics Board | Australia | HREC/16/Alfred/210  Local reference: 8/17,  SSA/17/Alfred/40-42 |
| Royal Prince Alfred Hospital Research Ethics Board | Australia | HREC/16/Alfred/210  Local reference: X17-140,  SSA/17/RPAH/203 |
| Western Health Research Ethics Board | Australia | HREC/16/Alfred/210  Local reference: n/a |
| Health Ethics Committee, Health Administration Regional Centre, Ministry of Health, Government of Portugal | Portugal | 09002117 |
| Ethics Committee, Health Sciences Research Unit, Higher Nursing School of Coimbra, Portugal | Portugal | P378-12/2016 |
| Central Hospital of Baixo Vouga, Entidades Públicas Empresariais, Aveiro, Portugal | Portugal | 777637 |
| National Health Service Research Ethics Board | United Kingdom | 17/NE/0035, 218161 |

**Table 2: Bootstrap Analysis**

| **Models** | **BBS** | | | | | **BESTest** | | | | |
| --- | --- | --- | --- | --- | --- | --- | --- | --- | --- | --- |
|  | **Variable** | **B** | **SE** | **95%CIs** | ***p*** | **B** | **SE** | **95%CIs** | ***p*** |  |
| **Functional impairment** | 30s CST | .69 | .16 | 0.42 - 1.04 | <.001^**^ | 1.52 | .30 | 0.96 - 2.14 | <.001^**^ |  |
|  | 6-MWT (meters) | .02 | .003 | 0.02 - 0.03 | <.001^**^ | .05 | .01 | 0.04 - 0.07 | <.001^**^ |  |
|  | R² = .41  Adj R² = .41  Overall *F (2,241) =84.9*  *P*-value <.001 | | | | | R² =.48  Adj R² = .48  Overall *F (2,241) =111.6*  *P*-value <.001 | | | | |
| **Clinical factors** | FEV_1_ | -.06 | .52 | -1.79 - 0.13 | .49 | .22 | .74 | -1.66 - 2.079 | .19 |  |
|  | Dyspnea (BDI) | 1.29 | .29 | 0.08 - 1.92 | <.001^**^ | 3.12 | .46 | 2.31 - 4.09 | <.001^**^ |  |
|  | Total comorbidities | -.53 | .16 | -0.87 - -0.18 | .004^*^ | -1.31 | .42 | -2.04 - -0.433 | <.002^*^ |  |
|  | Total medications | .13 | .17 | -0.19 - 0.459 | .47 | -.16 | .32 | -0.79 - 0.51 | .62 |  |
|  | Oxygen use | 3.49 | 1.22 | 1.16 - 5.95 | .011^*^ | 5.37 | 2.70 | 0.51 - 11.06 | .05 |  |
|  | Gait aid use | -5.44 | 1.75 | -8.95- -2.16 | <.005^*^ | -12.32 | 3.17 | -18.87 - -6.14 | <.001^**^ |  |
|  | Fall history last year | -1.58 | .93 | -3.3 - 0.28 | .1 | -5.09 | 2.09 | -9.26 - -1.23 | .01^*^ |  |
|  | R² = .31  Adj R² = .27  Overall *F (7.236) = 14.9*  *P*-value <.001 | | | | | R² =.38  Adj R² = .37  Overall *F (7,236) =20.1*  *P*-value <.001 | | | | |
| **Demographic and anthropometric factors** | Age | -.25 | .05 | -0.34 - 0.14 | <.001^**^ | -.68 | .11 | -0.87 - -0.45 | <.001^**^ |  |
|  | Sex | -2.35 | 1.07 | -4.31 - -0.140 | .03^*^ | -8.19 | 2.29 | -12.95 - -3.71 | <.001^**^ |  |
|  | BMI | -.08 | .07 | -0.21 - 0.05 | .34 | -.12 | .15 | -0.43 - 0.17 | .47 |  |
|  | Smoking history | -.002 | .02 | -0.02 - 0.04 | .87 | -.01 | .04 | -0.08 - 0.06 | .78 |  |
|  | R² = .10  Adj R² = .08  Overall *F (4,239) = 6.21*  *P*-value <.001 | | | | | R² = .15  Adj R² = .14  Overall F (4,239) *= 10.47*  *P*-value <.001 | | | | |
| **Full model** | 30s CST | .59 | .14 | 0.34 - 0.88 | <.001^**^ | 1.22 | .26 | 0.71 - 1.74 | <.001^**^ |  |
|  | 6-MWT (meters) | .02 | .003 | 0.01 - 0.03 | <.001^**^ | .04 | .01 | 0.02- 0.06 | <.002^*^ |  |
|  | FEV_1_ | -.24 | 1.35 | -3.84 - -0.12 | .38 | -.25 | 2.43 | -6.86 - -0.03 | .47 |  |
|  | Dyspnea (BDI) | .58 | .15 | 0.29 - 0.86 | .001^*^ | 1.55 | .34 | 0.89 - 2.18 | <.001^**^ |  |
|  | Total comorbidities | -.09 | .15 | -0.39 - 0.22 | .53 | -.28 | .38 | -0.98 - 0.50 | .48 |  |
|  | Total medications | .15 | .13 | -0.12 - 0.41 | .27 | -.13 | .25 | -0.65 - 0.31 | .63 |  |
|  | Oxygen use | 3.38 | .86 | 1.58 - 4.96 | .002^*^ | 5.52 | 2.09 | 1.23 - 9.47 | .01^*^ |  |
|  | Gait aid use | -4.25 | 1.44 | -7.23 - -1.36 | .006^*^ | -9.04 | 2.71 | -14.36 - -3.72 | .001^*^ |  |
|  | Fall history last year | -.87 | .76 | -2.22 - 0.63 | .28 | -3.17 | 1.63 | -6.30 - 0.20 | .06 |  |
|  | Age | -.01 | .06 | -0.13 - 0.10 | .82 | -.14 | .12 | -0.38 - 0.085 | .26 |  |
|  | Sex | .87 | .99 | -1.47 - 2.39 | .44 | -.30 | 2.07 | -5.44 - 2.72 | .89 |  |
|  | BMI | -.04 | .06 | -.136,.100 | .53 | -.05 | .14 | -0.28 - 0.25 | .76 |  |
|  | Smoking history | .02 | .01 | 5.91- 0.04 | .06 | .04 | .02 | -.02 - 0.08 | .09 |  |
|  | R² = .51  Adj R² = .48  Overall *F (13,230) = 18.39*  *P*-value <.001 | | | | | R² = .58  Adj R² = .55  Overall *F (13,230) =24.05*  *P*-value <.001 | | | | |
| ***** P <0.05, **P < 0.01 (N = 244); *B:* unstandardized coefficient, *SE*: standard error, *β*: standardized coefficient, BBS: Berg Balance Scale, BESTest : Balance Evaluation Systems Test, FEV_1_: forced expiratory volume in 1 second, BDI: Baseline Dyspnea Index, BMI: body mass index, 30s CST: 30-second Repeated Chair Stand Test, 6-MWT: Six-Minute Walk Test. | | | | | | | | | | |

**Table 3: Description and Interpretation of Balance Scoring Systems**

| Measure | Description | Scoring Range | Interpretation |  |
| --- | --- | --- | --- | --- |
| **Berg Balance Scale (BBS)** | - 14 functional tasks such as reaching, standing, turning, and transfers. - Each item is scored from 0 (unable to perform) to 4 (independent). - The total score reflects overall balance ability. - It takes 15 to 20 minutes to administer - Age: (adult 18-64), elderly adult (64+) | 0-56 | Higher scores indicate better balance. Scores **below 46** suggest increased fall risk, and scores **below 40** indicate high fall risk in older adults and individuals with COPD. MCID = 5–7 points; MDC = 3.5–5.9 points | 1. Berg K, Wood-Dauphine S, Williams JI, Gayton D. Measuring balance in the elderly: preliminary development of an instrument. Physiotherapy Canada. 1989;41(6):304-11. 2. Beauchamp MK, Harrison SL, Goldstein RS, Brooks D. Interpretability of Change Scores in Measures of Balance in People With COPD. Chest. 2016;149(3):696-703. 3. Jácome C, Cruz J, Oliveira A, Marques A. Validity, reliability, and ability to identify fall status of the Berg Balance Scale, BESTest, Mini-BESTest, and Brief-BESTest in patients with COPD. Physical therapy. 2016 Nov 1;96(11):1807-15. 4. Beauchamp MK, Harrison SL, Goldstein RS, Brooks D. Interpretability of Change Scores in Measures of Balance in People With COPD. Chest. 2016;149(3):696-703. 5. https://www.sralab.org/rehabilitation-measures/berg-balance-scale |
| **Balance Evaluation Systems Test (BESTest)** | - 36-item assessment of balance impairments across 6 postural control domains: 1) Biomechanical constraints 2) Stability limits/verticality 3) Anticipatory postural adjustments 4) Postural responses 5) Sensory orientation 6) Stability in gait - Item-level scores range from 0 (severe impairment) to 3 (no impairment). - It takes 20-30 minutes. - Age (65+) | - 0–108 (raw score), converted to (0 **-100%)** - Also, total sub-scores exist for each of the postural control domains**.** | Higher scores indicate better balance. Scores **below 69%** (equivalent to 82 points) are associated with increased fall risk. MCID = 13–17 points; MDC = 6.3 points. | 1. Horak FB, Wrisley DM, Frank J. The balance evaluation systems test (BESTest) to differentiate balance deficits. Physical therapy. 2009;89(5):484-98. 2. Marques A, Almeida S, Carvalho J, Cruz J, Oliveira A, Jácome C. Reliability, Validity, and Ability to Identify Fall Status of the Balance Evaluation Systems Test, Mini&#x2013;Balance Evaluation Systems Test, and Brief&#x2013;Balance Evaluation Systems Test in Older People Living in the Community. Archives of Physical Medicine and Rehabilitation. 2016;97(12):2166-73.e1. 3. Jácome C, Cruz J, Oliveira A, Marques A. Validity, reliability, and ability to identify fall status of the Berg Balance Scale, BESTest, Mini-BESTest, and Brief-BESTest in patients with COPD. Physical therapy. 2016 Nov 1;96(11):1807-15. 4. https://www.sralab.org/rehabilitation-measures/balance-evaluation-systems-test |
